# Laboratory indicators of hypothyroidism and TgAA-positivity in the Eurasian dog breed

**DOI:** 10.1371/journal.pone.0280906

**Published:** 2023-01-24

**Authors:** Martina Schlipf, Andrea Fischer, Martina Patzl, Katrin Hartmann, Alexander Pankraz, Martina Dick, Yury Zablotski, Helmut Küchenhoff, Astrid Wehner

**Affiliations:** 1 Clinic of Small Animal Medicine, Centre for Clinical Veterinary Medicine, LMU Munich, Munich, Germany; 2 Institute of Immunology, Department of Pathobiology, University of Veterinary Medicine, Vienna, Austria; 3 Biocontrol, Bioscientia Healthcare GmbH, Ingelheim, Germany; 4 Institute of Statistics, LMU Munich, Munich, Germany; Universidade Federal de Minas Gerais, BRAZIL

## Abstract

**Background:**

Hereditary hypothyroidism represents a concern for dog breeders; thus, surveillance programs have been established for several dog breeds.

**Methods:**

Thyroid profiles (total thyroxine (TT4), thyrotropin (thyroid stimulating hormone (TSH)), and thyroglobulin autoantibodies (TgAA)) collected as part of a breed surveillance program in Eurasians (2009–2017) were retrospectively analyzed. The study included data from 1,501 Eurasians from a German breeding club. Classification was exclusively based on laboratory data. Hypothyroidism was defined as a combined decrease in TT4 and increase in TSH in serum and was classified as TgAA-positive and TgAA-negative hypothyroidism. Thyroglobulin autoantibodies (TgAA) independent of the concentrations of TT4 and TSH were determined. The overall prevalence of hypothyroidism, TgAA-positive hypothyroidism, TgAA-negative hypothyroidism and TgAA-positivity was assessed when the dogs entered the program. Follow-up laboratory data was available for 324 dogs without hypothyroidism on initial examination.

**Results:**

The initial screening was performed at a median age of 18 months (interquartile range (IQR): 15–29). The overall prevalence of hypothyroidism was 3.9% (n = 58; 95% CI: 2.9–4.8%) and the prevalence of a positive TgAA status was 7.9% (n = 118; 95% CI: 6.6–9.3%). The prevalence of TgAA-positive and TgAA-negative hypothyroidism was 1.7% (n = 26; 95% CI: 1.1–2.4%) and 2.1% (n = 32; 95% CI: 1.4–2.9%), respectively. 22.0% of dogs with positive TgAA status (26/118) were already hypothyroid on initial examination. Overall, 42.5% (17/40) of TgAA-positive dogs on initial examination developed hypothyroidism on follow-up.

**Conclusion:**

The results of this study demonstrate that the Eurasian dog breed exhibits a relevant risk for hypothyroidism and presence of TgAA. The predictive value of TgAA for hypothyroidism or developing hypothyroidism was high in this breed. Further investigations with longitudinal studies in individual dogs are warranted.

## Introduction

Increasing evidence indicates that the prevalence of hypothyroidism is particularly high in some dog breeds and that a hereditary component is involved in the pathogenesis at least in some of the breeds [1–4].

The Eurasian is a relatively new breed and was first bred in Germany. Initially, Wolf Spitz (Keeshond) and Chow Chow were mated, and Samoyed was crossed thereafter. The breed was recognized in 1973 by the Fédération Cynologique Internationale [5] and the name of the breed symbolizes their combined European and Asian heritage. Since 2009, the largest breeding club of Eurasians in Germany and worldwide [6] has identified several dogs affected by hypothyroidism and implemented a surveillance program. Regarding the Eurasian’s ancestral breeds, there are some hints to a possible genetic basis for thyroid disease. To the authors’ knowledge, the Chow Chow does not exhibit a predisposition for hypothyroidism. Similarly, the Keeshond is also not widely recognized as a breed primarily affected by this disease; however, a study by Bellumori *et al*. (2013) listed this breed in the top 5 breeds affected by hypothyroidism in a Californian veterinary hospital population, with a prevalence of 6.6% based on the hospital’s electronic records [7]. Samoyeds were found to have a prevalence of 8.8% (n = 3/35) in a recent study in an Indian veterinary hospital population [8].

In humans, Hashimoto’s thyroiditis, a chronic autoimmune inflammation of the thyroid gland, represents the most common cause of hypothyroidism and is the most frequent autoimmune disease [9, 10]. Women have a higher risk than men [9, 11]. Clinically, the thyroid gland enlarges regardless of whether hypothyroidism has already manifested. Histologically, lymphocytic infiltration of the thyroid is present [9]. Lymphocytes come in close contact with thyrocytes and are believed to be mediators of thyrocyte destruction [9]. Autoantibodies against thyroid antigens (thyroid peroxidase autoantibodies and thyroglobulin autoantibodies (TgAA)) are typically present; however, the role of these antibodies in the disease remains unclear. These circulating antibodies are considered the best serum marker for the diagnosis of Hashimoto’s thyroiditis [9]. Antibodies against thyroid peroxidase are the most prevalent, but TgAA precede their appearance and indicate the initial phases of an immune response. Antibodies can occur years before the onset of hypothyroidism [9]. Hashimoto’s thyroiditis is thought to arise from a combination of genetic susceptibility, epigenetic effects, and various environmental triggers (e.g., dietary iodine content, infections, and pregnancy) [11, 12].

The pathogenesis of canine lymphocytic thyroiditis (CLT) is believed to be comparable to that of Hashimoto’s thyroiditis [4, 13, 14]. Hypothyroidism in dogs is assumed to be a primarily immune-mediated disease [15, 16] that results from defective immune regulation [13, 17]. Although the destruction of canine thyroid tissue can be fully explained by direct T-cell toxicity, autoantibodies are also thought to be important in the pathogenesis of CLT [18, 19]. In dogs, TgAA are found most frequently, followed by antibodies against the thyroid hormones thyroxine (T4) and triiodothyronine (T3) [3, 13, 20]. Antibodies against thyroid peroxidase seem to play a minor role in dogs [21]. Autoantibody-positive hypothyroid individuals have been identified in several dog breeds [1–3]. One study has shown that at least 50% of dogs affected by hypothyroidism suffer from CLT based on TgAA measurements [13]. Lymphocytic thyroiditis in dogs is characterized by lymphocytes, plasma cells, and macrophages infiltrating the thyroid gland, resulting in the destruction of thyroid follicles and replacement with fibrous connective tissue [22, 23]; however, goiter has not yet been recognized [13]. The progression from the occurrence of TgAA to the manifestation of hypothyroidism in dogs encompasses different stages [3, 13], which is similar to that noted in humans. In the first stage, which has been classified as silent thyroiditis, TgAA are present in sera, but parameters of thyroid function such as total thyroxine (TT4), free thyroxine (fT4) and thyrotropin (thyroid stimulating hormone (TSH)) remain normal. During this first stage, lymphocytic infiltration of the thyroid gland develops. The second stage is called subclinical hypothyroidism. To compensate for the beginning of thyroid dysfunction, the secretion of TSH from the pituitary gland increases, and the TSH level is elevated. Thyroglobulin autoantibodies (TgAA) are present and the circulating TSH concentration is increased, but TT4 and fT4 remain within the reference range. In the third stage, TgAA-positive hypothyroidism develops and the physiological architecture of the thyroid lobes is gradually lost. Affected dogs have low TT4 and fT4 concentrations, while TSH concentrations typically remain elevated. Various TgAA titers are noted, and clinical signs of hypothyroidism are frequently present. The fourth and final stage is called noninflammatory atrophic hypothyroidism or TgAA-negative hypothyroidism and is probably linked to the disappearance of TgAA and atrophy of the thyroid gland. At this point, the fT4 and TT4 levels are low, and the TSH levels remain elevated or are starting to decrease. The time period from thyroiditis to antibody-negative hypothyroidism is reported to range between one and two years but is variable. Progression through all stages may not necessarily occur, and clinical hypothyroidism might not be reached in all cases [3, 13, 24]. Therefore, TgAA provide evidence that CLT is present but do not impart information on thyroid function per se.

The objectives of this study were to calculate the overall prevalence of laboratory indicators of hypothyroidism (a total T4 (TT4) concentration below reference interval (RI) combined with a TSH concentration above RI) and TgAA in a population of Eurasians participating in a breed surveillance program. The secondary aim was to evaluate a possible association with sex and to provide follow-up evaluations of dogs, which were retested later.

## Methods

### Ethical approval

Ethical approval was obtained from the Ethics Committee of the Faculty of Veterinary Medicine, LMU Munich, Germany (accession number 161-13-3-2019). Informed written consent was obtained from the breeding club, which had obtained informed verbal consent from their members. Personal data of dog owners were deleted and only anonymized data were provided for analysis.

### Breed surveillance program of the Eurasian club

**Fig 1:**
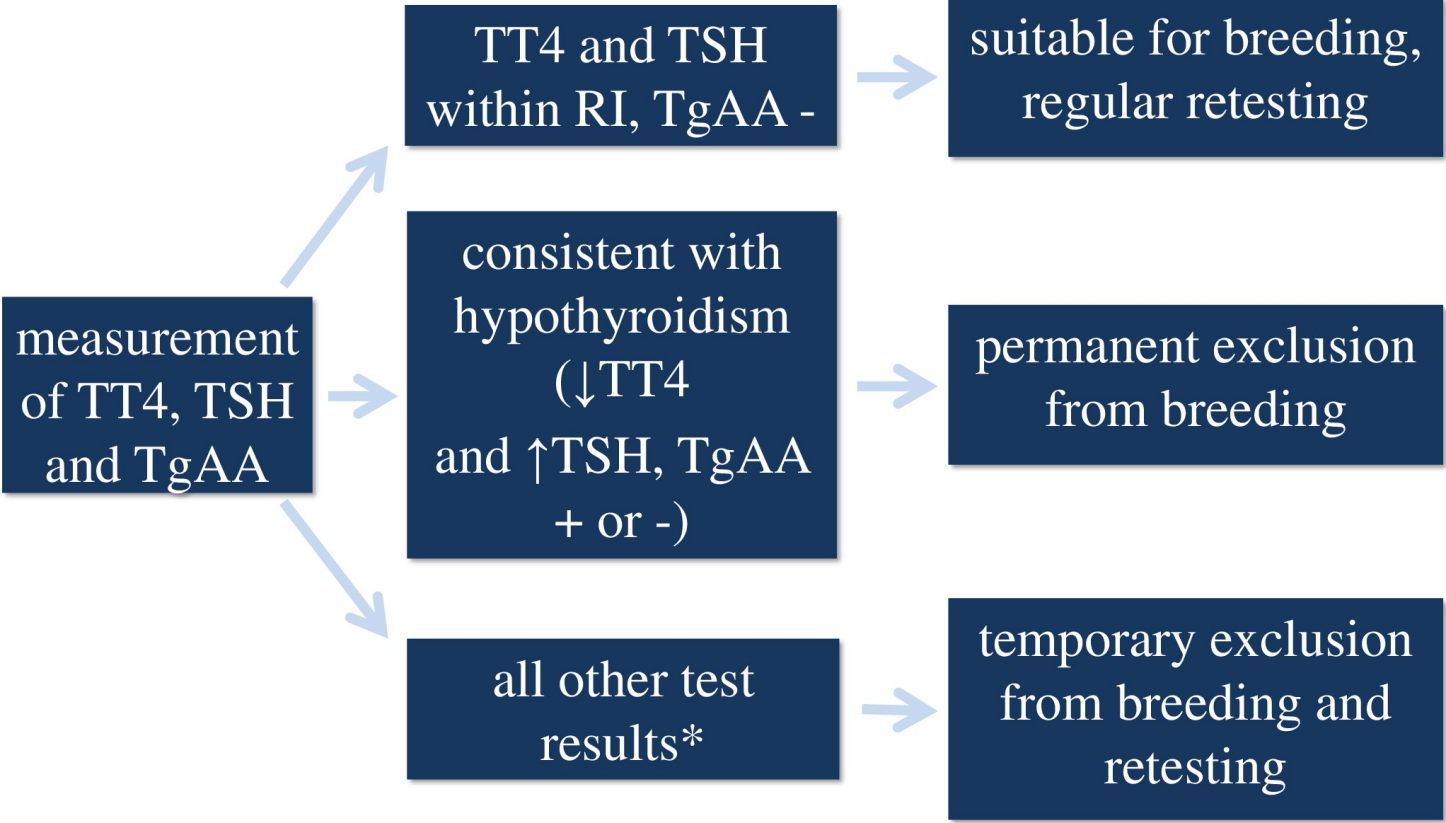
Thyroid screening scheme in Eurasian dogs participating in the screening program. TT4: total thyroxine concentration; TSH: thyrotropin concentration; TgAA: presence of thyroglobulin autoantibodies; RI: reference interval; +: positive; -: negative; ↑: greater than RI of the laboratory; ↓: less than RI of the laboratory. * See classification/characteristics of groups 3, 4, 5 and 6.

Thyroid testing (TT4, TSH and TgAA) was implemented by a German Eurasian breeding club (Kynologische Zuchtgemeinschaft Eurasier e.V.). For the study, data obtained between January 1, 2009, and December 31, 2017, was analyzed retrospectively. Participation in this program was mandatory for all Eurasians currently involved in breeding and those which would be intended for breeding at a later time point; however, Eurasians that were not used for breeding were also allowed to participate. If breeding was desired, the first testing for TT4, TSH and TgAA was recommended at the age of 12–16 months, and retesting was advised within the first 3 years of life and before mating. Retesting was also encouraged when thyroid testing revealed any abnormalities (see “all other results” in Fig 1). Retesting for TgAA was recommended if a dog had previously tested TgAA negative; however, the extent to which dogs were involved depended on the owners of the participating dogs. Only clinically healthy dogs with no known diseases were allowed to participate. Their health status was assessed with a questionnaire from the breeding club in which the owners had to respond to questions about any past or current diseases or medications, changes in behavior or temperament and haircoat or skin changes noticed in their dogs. To minimize any effects of vaccination, the breeding club recommended collecting the blood samples not at the time when the dog was vaccinated. New dogs were continuously included in the thyroid screening program by the breeding club, and some of the dogs were tested repeatedly over the years. Screening of the participating dogs was continued until a dog developed hypothyroidism, was affected by another chronic disease, or the owners chose to withdraw from the program as they did not want to continue with the breeding process.

### Laboratory analysis

All serum samples from dogs that were involved in the breeding program were sent to a diagnostic laboratory that participated in the Quality Assurance Scheme of the European Society of Veterinary Endocrinology (Biocontrol, Ingelheim, Germany). A detailed description of the applied methods and reference ranges is provided below:

Total T4 (TT4) was analyzed using a chemiluminescence enzyme immunoassay (CLEIA) based on the Immulite Canine T4 assay on an Immulite 2000 test system according to the manufacturer’s specification (Siemens Healthineers, Erlangen, Germany). Total T4 (TT4) concentrations were converted from μg/dl to nmol/l by multiplying the μg/dl value by 12.87. This test has been frequently used to measure TT4 in dogs and is incorporated in the quality assurance scheme of the European Society of Veterinary Endocrinology [25]. In addition, this test has been compared to the validated Immulite 1000 TT4 assay and a strong correlation of results was demonstrated (r = 0.99) [26]. A recent study compared test results of the Immulite 2000 TT4 assay to a validated enzyme immunoassay (EIA) [27] and found an excellent correlation of both tests (r = 0.99). Bland-Altman plot showed a good agreement of both tests. A proportional bias was only noted at high concentrations (>77.2 nmol/l) [28]. The lower and upper limits of detection were 6.4 nmol/l and 193.1 nmol/l, respectively. Values greater than 154.5 nmol/l were also analyzed after dilution to confirm the results and to exclude the possibility that a value is beyond the linear measurement range. The intra-assay variation was 7.7% at 20.7 nmol/l. The results reported as <6.4 nmol/l were changed to 6.4 nmol/l for statistical evaluation. The laboratory reference range for TT4 was 19.3–51.5 nmol/l.

Thyrotropin (TSH) was measured using a chemiluminescence enzyme immunoassay (CLEIA) with the Canine TSH Kit on an Immulite 2000 test system according to the manufacturer’s specifications (Siemens Healthineers, Erlangen, Germany) [29–40]. The lower and upper limits of detection were 0.03 ng/ml and 12 ng/ml, respectively. Samples with values greater than 8 ng/ml were analyzed after dilution to confirm the result and to exclude the possibility that a value is beyond the linear measurement range. The intra-assay coefficients of variation ranged from 3.4% at 2.04 ng/ml to 4.5% at 0.48 ng/ml. The results reported as <0.03 ng/ml were changed to 0.03 ng/ml for statistical evaluation. The reference range of the laboratory was <0.3 ng/ml, and a range of 0.3 to <0.5 ng/ml was assessed as inconclusive. For the purpose of this study, we considered only values ≥0.5 ng/ml as increased.

Autoantibodies directed against canine thyroglobulin were measured using an in-house ELISA. The validation of this assay in dogs has been described in detail elsewhere [41] and the present protocol included two modifications. For the estimation of individual serum background (to exclude false-positive results), biotinylated (succinimidyl-6-(biotinamido) hexanoate) canine thyroglobulin was replaced by assay buffer in corresponding wells on the same plate. A pool of negative canine sera was used for the calculation of the threshold (180% of the mean) and was analyzed 8 times; all other samples were analyzed in duplicate. Sera with a background optical density greater than the threshold were designated as not assessable. For the purpose of this study, we only considered if TgAA were present (positive) or not (negative).

### Data processing

The breeding club entered all laboratory data into the software “Breedmaster” (Breedmaster–Pedigreedatenbank, Hilpoltstein, Germany) together with the name of the dog, its identification number (ID) given by the breeding club, date of birth, sex, names and IDs of the sire and dam, and their dates of birth. Additionally, the date of each laboratory examination, the overall condition of the dog (based on the owners’ answers to the breeding club’s questionnaire) and whether thyroid replacement therapy had been started by the owner were recorded.

Datasets were exported to Microsoft Excel (Microsoft Office, 2009) by the breeding club, and the anonymized data was then forwarded to the authors for data analysis. Datasets were reviewed, and data cleaning was performed. The data cleaning process involved the removal of incomplete datasets and any results which could be affected by thyroid hormone supplementation, which was evident from comments inserted by the breeding club. For the calculation of prevalence of hypothyroidism and TgAA, only data from the first examination of a dog was considered. If data from follow-up examinations were available, this data was analyzed separately. Follow-up examinations were evaluated from dogs which were not hypothyroid on initial examination to observe disease progression. For this analysis, the last available laboratory result of these dogs was included.

Based on the laboratory results of TT4, TSH and TgAA measurements, dogs were classified into different groups which were formed according to the progression of stages from lymphocytic thyroiditis to overt thyroid functional failure as previously described by Graham *et al*. [3, 13]:

- hypothyroidism (group 1): a combination of a TT4 concentration less than the RI and a TSH concentration greater than the RI
- TgAA-positive hypothyroidism (group 1a): dogs of group 1 with a positive TgAA status
- TgAA-negative hypothyroidism (group 1b): dogs of group 1 with a negative TgAA status
- euthyroidism (group 2): a combination of a TT4 and TSH concentration within their respective RIs and a negative TgAA status
- subclinical hypothyroidism (group 3): a combination of a TT4 concentration within the RI, a TSH concentration greater than the RI and a positive TgAA status
- TgAA-positive dogs with unremarkable thyroid function (group 4): a combination of TT4 and TSH concentrations within their respective RIs and a positive TgAA status
- potential nonthyroidal illness (group 5): a combination of a TT4 concentration less than the RI, a TSH concentration within the RI and a negative TgAA status
- unclassified (group 6): a combination of a TT4 concentration within the RI, a TSH concentration greater than the RI and a negative TgAA status; a combination of a TT4 concentration greater than the RI, a TSH concentration within or greater than the RI and a negative or positive TgAA status; or a combination of a TT4 concentration less than the RI, a TSH concentration within the RI and a positive TgAA status

### Statistical analysis

The R 4.0.3 statistical package (R Core Team, 2020) was used for statistical analysis.

Prevalences were calculated based on the Wald method, including a 95% confidence interval. The normality of the data distribution was tested using the Shapiro‒Wilk test, and nonparametric tests were used, as most data was not normally distributed. Groups were compared using the Kruskal‒Wallis test. Partial epsilon squared was calculated to assess the impact of the groups on age, TT4 and TSH, and Dunn pairwise test was performed as a post hoc test. To determine whether gender and groups are dependent on each other, p values as well as the V Cramer, Bayesian V Cramer and Bayes factor were calculated. The confidence level was 0.95. For the analysis of the follow-up examinations, only descriptive statistical methods were used.

## Results

A total of 2,077 datasets were exported to Microsoft Excel (Microsoft Office, 2009). After data cleaning, thyroid screening results of 1,501 Eurasians (770 female, 731 male) were evaluated. Dogs entered the screening program at a median age of 18 months (range: 8–160 months). The median TT4 concentration was 27.0 nmol/l (interquartile range (IQR): 20.6–34.8 nmol/l). The median TSH concentration was 0.09 ng/ml (IQR: 0.05–0.15 ng/ml). Follow-up examinations were available from 324 dogs.

### Prevalence

Overall, 3.86% of Eurasians were hypothyroid on initial testing and 118/1,501 dogs (groups 1a, 3, and 4 as well as 8 dogs in group 6) tested positive for TgAA, leading to a prevalence of TgAA of 7.86% (95% CI: 6.55–9.34%) in the entire population. Altogether, 74.22% of Eurasians had unremarkable thyroid test results. Detailed data of the different groups (groups 1–6) is presented in Table 1.

**Table 1:** Prevalence of hypothyroidism (group 1), subclinical hypothyroidism (group 3) and TgAA-positivity with unremarkable thyroid function (group 4) in Eurasian dogs (N = 1,501) on initial screening.

Group	Characteristics	n	Prevalence (%)	Lower 95% CI	Upper 95% CI
Hypothyroidism (group 1)	↓TT4, ↑TSH	58	3.86	2.89%	4.84%
TgAA-positive hypothyroidism (group 1a)	↓TT4, ↑TSH, TgAA pos.	26	1.73	1.07%	2.39%
TgAA-negative hypothyroidism (group 1b)	↓TT4, ↑TSH, TgAA neg.	32	2.13	1.4%	2.86%
Euthyroidism (group 2)	= TT4, = TSH, TgAA neg.	1,114	74.22	72%	76.43%
Subclinical hypothyroidism (group 3)	= TT4, ↑TSH, TgAA pos.	11	0.73	0.3%	1.16%
TgAA-positive dogs with unremarkable thyroid function (group 4)	= TT4, = TSH, TgAA pos.	73	4.86	3.78%	5.95%
Potential nonthyroidal illness (group 5)	↓TT4, = TSH, TgAA neg.	190	12.66	10.98%	14.34%
Unclassified (group 6)	all other combinations	55	3.66	2.71%	4.61%

↑: greater than the RI of the laboratory; ↓: less than the RI of the laboratory; =: within the RI of the laboratory

Of the 55 unclassified dogs (group 6), 19 had a TSH concentration greater than the RI, 28 had a TT4 concentration greater than the RI, 2 dogs had a TT4 concentration greater than the RI and a positive TgAA status, and 6 dogs had a TT4 concentration less than the RI and a positive TgAA status.

The calculated prevalence of hypothyroidism of 3.9% represents the lowest possible prevalence in this population because 25% of hypothyroid dogs have a TSH concentration within the RI [42–44]. Thus, the 58 hypothyroid dogs (group 1) in this study may only represent 75% of the actual total number; the other 25% (19 dogs) may contribute to this actual hypothyroid group, resulting in a total of 77/1,501 (5.1%) hypothyroid dogs.

### Group characteristics

#### Age

**Fig 2:**
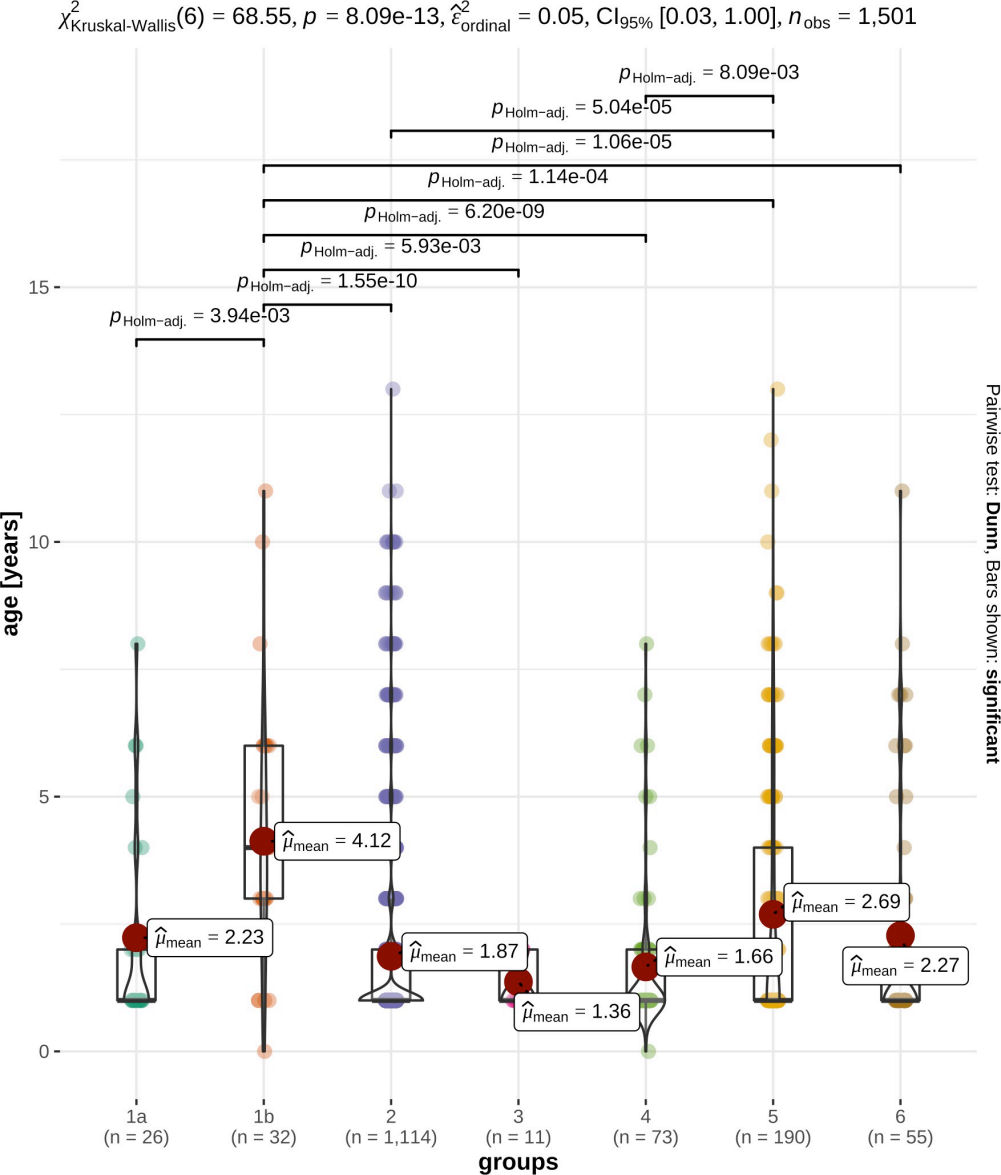
Box and violin plots illustrate the distribution of age in the different groups. TgAA-positive hypothyroidism (group 1a), TgAA-negative hypothyroidism (group 1b), euthyroidism (group 2), subclinical hypothyroidism (group 3), TgAA-positive dogs with unremarkable thyroid function (group 4), potential nonthyroidal illness (group 5), unclassified dogs (group 6). The x-axis shows the different groups, and the y-axis represents the age of the dogs in years. The p value of 8.09e-13 (p<0.001) (in the first line of the graphic) provides very strong evidence that at least one of the groups is different from the others [45]. However, partial epsilon squared (${\hat{\varepsilon }}_{ordinal}^{2}$) with a 95% CI as the measure of the effect size for Kruskal‒Wallis test is 0.05. This finding indicates that the effect of the groups on the age distribution is minimal [45]. Using Dunn pairwise tests, the displayed p values illustrate the groups between which a significant difference exists [45]. A significant difference in the age distribution was noted in group 1b (TgAA-negative hypothyroidism) compared with all the other groups (1b/1a, 1b/3, p<0.01; 1b/2, 1b/4-6, p<0.001), between group 5 (potential nonthyroidal illness) and groups 2 (p<0.001) and 4 (p<0.01).

Dogs with TgAA-negative hypothyroidism (group 1b) were significantly older than dogs of all other groups (Table 2, Fig 2). Also, dogs with potential nonthyroidal illness (group 5) were older than dogs with isolated TgAA positivity (group 4) and euthyroid dogs (group 2) (Fig 2).

**Table 2:** Age of the dogs, TT4 and TSH concentrations in the different groups. The data is provided as median and IQR if not normally distributed, and as mean and SD (*) if normally distributed.

Group	Age (years) Median (IQR)	TT4 (nmol/l) Median (IQR)	TSH (ng/ml) Median (IQR)
1: Hypothyroidism	3.000 (3.750)	7.080 (6.114)	2.565 (2.473)
1a: TgAA-positive hypothyroidism	1.000 (1.000)	9.654 (8.367)	2.495 (2.553)
1b: TgAA-negative hypothyroidism	4.000 (3.000)	6.436 (4.183)	2.675 (2.212)
2: Euthyroidism	1.000 (1.000)	29.606 (11.585)	0.090 (0.080)
3: Subclinical hypothyroidism	1.000 (1.000)	25.276 (5.093)*	1.370 (0.540)
4: TgAA-positive dogs with unremarkable thyroid function	1.000 (1.000)	28.865 (7.651)*	0.090 (0.090)
5: Potential nonthyroidal illness	1.000 (3.000)	15.446 (5.149)	0.070 (0.098)
6: Unclassified	1.000 (1.000)	52.775 (37.972)	0.090 (0.540)

TT4 reference interval: 19.308–51.488 nmol/l; TSH reference interval: <0.5 ng/ml. IQR: interquartile range; SD: standard deviation.

#### Sex

**Fig 3:**
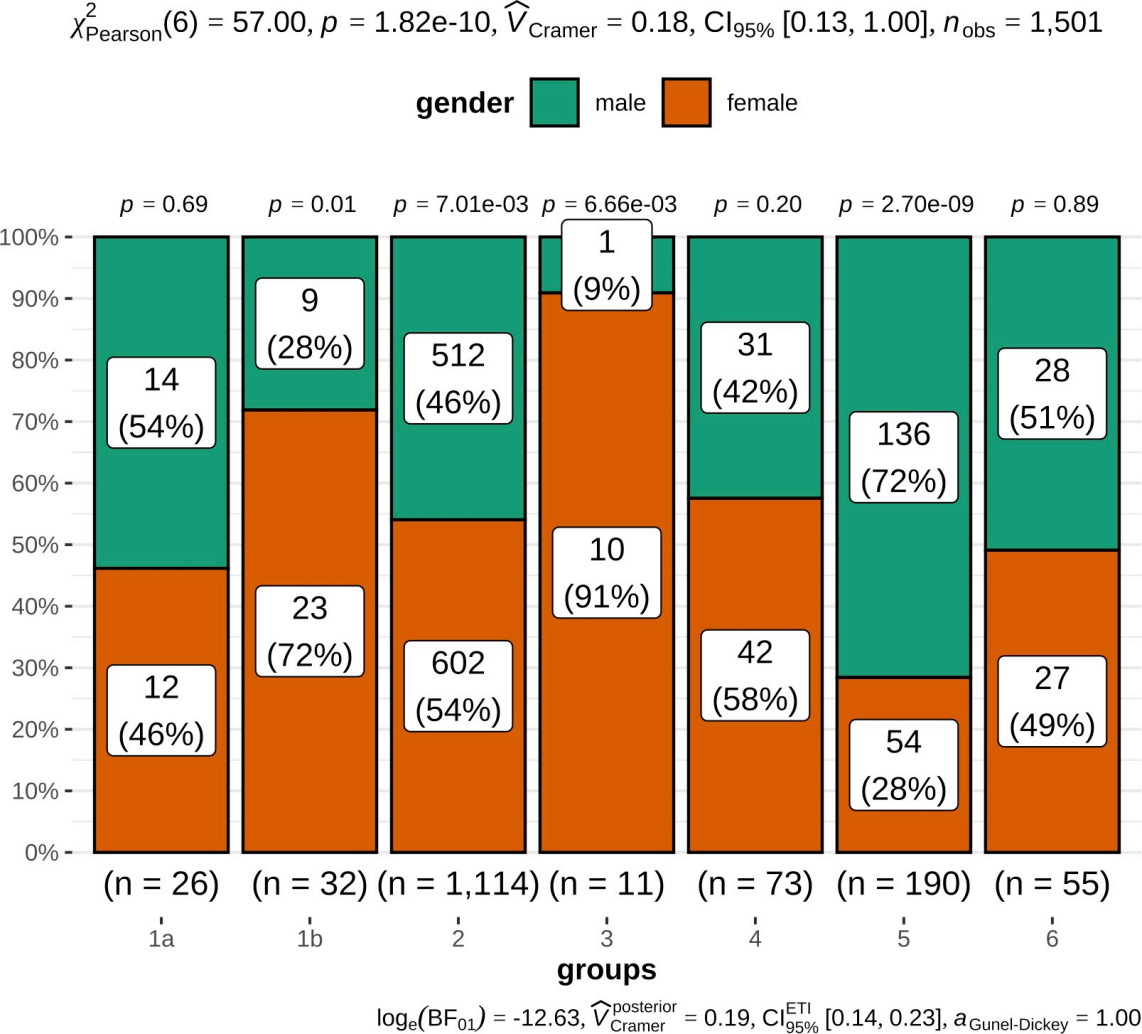
Stacked bar charts show the proportions of female and male dogs in the different groups. TgAA-positive hypothyroidism (group 1a), TgAA-negative hypothyroidism (group 1b), euthyroidism (group 2), subclinical hypothyroidism (group 3), TgAA-positive dogs with unremarkable thyroid function (group 4), potential nonthyroidal illness (group 5), unclassified dogs (group 6). The groups are displayed on the x-axis, and percentages are displayed on the y-axis. Absolute numbers and percentages are displayed in each category. The p value of <0.001 indicates that gender and groups are dependent on each other; however, the V Cramer value of 0.18 with its 95% CI as the effect size next to the p value indicates only a weak association [46]. The Bayesian V Cramer effect size (${\hat{V}}_{Cramer}^{posterior}$) with its 95% highest density intervals of 0.20 also only shows a mild to moderate association [46]. In contrast, the Bayes factor (log_e_(BF_01_)) indicates decisive evidence for an association [46]. Proportion tests in each group show that the proportions of females and males differ significantly in groups 1b, 2, 3 (p≤0.01) and 5 (p<0.001).

Females were significantly overrepresented in TgAA-negative hypothyroidism (group 1b), euthyroidism (group 2), and subclinical hypothyroidism (group 3); whereas in potential nonthyroidal illness (group 5), more males were affected (p values see Fig 3).

#### TT4

**Fig 4:**
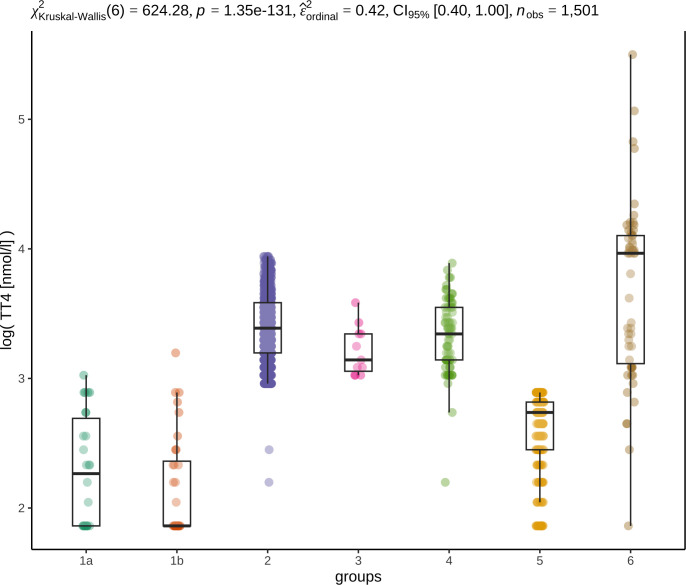
Box and dot plots illustrate the distribution of TT4 concentrations in the different groups. TgAA-positive hypothyroidism (group 1a), TgAA-negative hypothyroidism (group 1b), euthyroidism (group 2), subclinical hypothyroidism (group 3), TgAA-positive dogs with unremarkable thyroid function (group 4), potential nonthyroidal illness (group 5), unclassified dogs (group 6). The different groups are shown on the x-axis, and logarithmical TT4 values in the unit nmol/l are shown on the y-axis. At least one of the groups differs from the other groups, as suggested by the p value of 1.35e-131 (p<0.001) displayed in the first line of the graphic [45]. Partial epsilon squared (${\hat{\varepsilon }}_{ordinal}^{2}$) with a 95% CI as the measure of the effect size for Kruskal‒Wallis test is 0.42 and demonstrates that the effect of the groups on the distribution of TT4 concentrations is large [45]. Dunn pairwise test was used as a post hoc test. A significant difference was found in the distribution of TT4 concentrations in group 1a (1a/2, 1a/4, 1a/6, p<0.001; 1a/3, p<0.01), group 1b (1b/2, 1b/4, 1b/6, p<0.001; 1b/3 p<0.01) and group 5 (5/2, 5/4, 5/6, p<0.001; 5/3, p<0.01) compared with all the other groups, but not with each other, and group 6 was significantly different from all the other groups (6/1a, 6/1b, 6/5, p<0.001; 6/4, p<0.01; 6/2, 6/3, p<0.05).

The TT4 concentrations were significantly lower in hypothyroid dogs (groups 1a, 1b) and dogs with potential nonthyroidal illness (group 5) than in all other groups. Total T4 concentrations above RI were only documented in a few unclassified dogs (group 6) (Fig 4).

#### TSH

**Fig 5:**
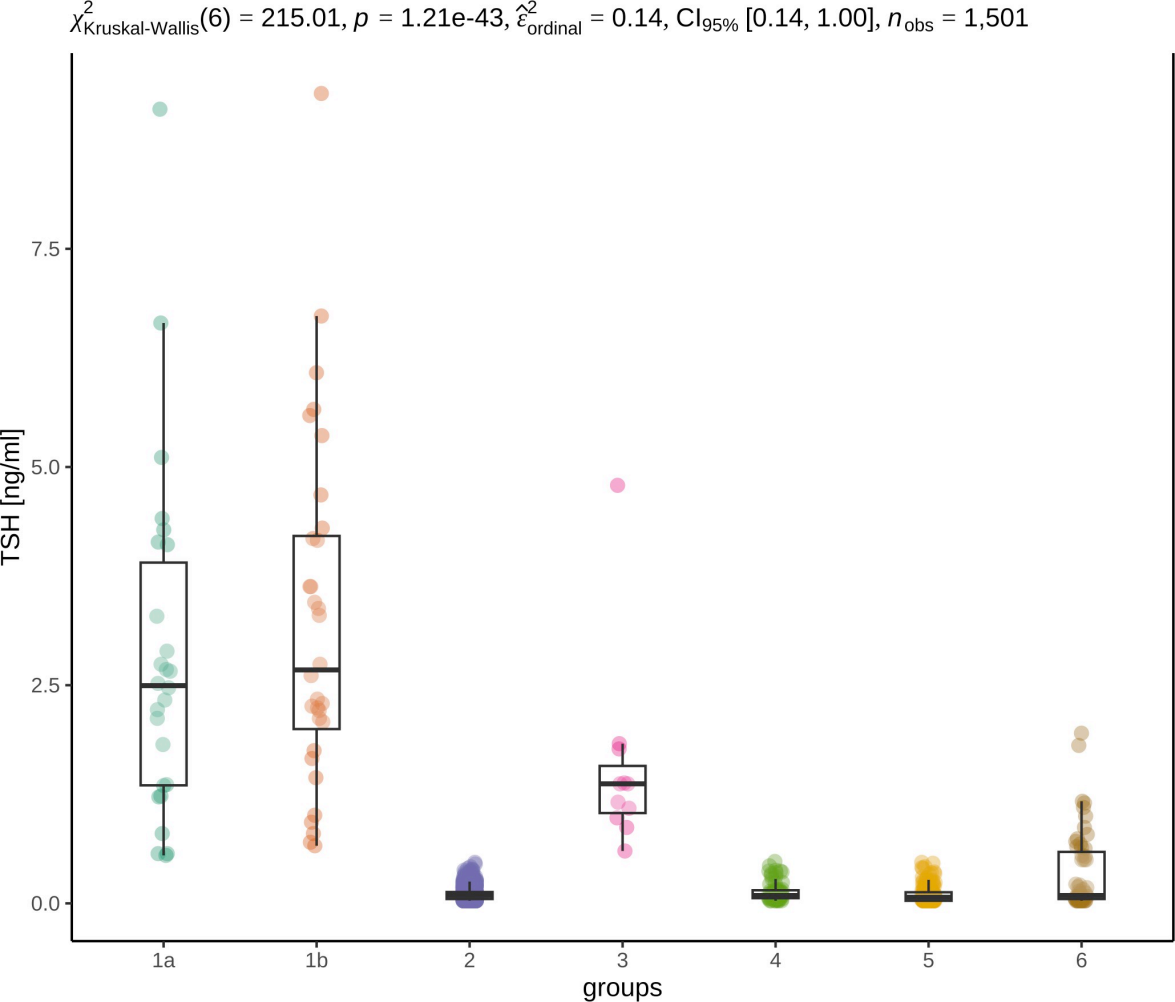
Box and dot plots show the distribution of TSH concentrations in the different groups. TgAA-positive hypothyroidism (group 1a), TgAA-negative hypothyroidism (group 1b), euthyroidism (group 2), subclinical hypothyroidism (group 3), TgAA-positive dogs with unremarkable thyroid function (group 4), potential nonthyroidal illness (group 5), unclassified dogs (group 6). The groups are displayed on the x-axis, and the y-axis shows the TSH concentrations of the dogs in ng/ml. The p value of 1.21e-43 (p<0.001) (in the first line of the graphic) provides very strong evidence that at least one of the groups differs from the others [45]. Partial epsilon squared (${\hat{\varepsilon }}_{ordinal}^{2}$) with a 95% CI as the measure of the effect size for Kruskal‒Wallis test is 0.14, indicating that the effect of the groups on the distribution of TSH concentrations is large [45]. P values were determined by Dunn pairwise tests. A significant difference in the distribution of TSH concentrations was observed in groups 1a, 1b and 3 compared with all the other groups (p<0.001 in all cases), but not among each other, and between groups 5 and 2/4/6 (p≤0.01).

TSH concentrations were significantly higher in hypothyroid dogs (groups 1a, 1b) and dogs with subclinical hypothyroidism (group 3) than in all other groups, whereas dogs with potential nonthyroidal illness (group 5) had the lowest TSH concentrations (Fig 5).

Descriptive statistics are provided in detail for each group in Table 2.

### Follow-up examinations

Testing was repeated in 324 out of 1,501 (21.6%) dogs (179 females, 145 males) which were not hypothyroid on initial examination. The median age at the first examination was 19 months (range: 12–111 months). The median time span between the first and the last examination was 733 days (range: 12–3,019 days). The median age at the last examination was 51 months (range: 14–166 months). The number of re-examinations varied from one to five. Most dogs received only one re-examination (220 dogs). The other dogs received 2 (60 dogs), 3 (38 dogs), 4 (5 dogs) and 5 (1 dog) re-examinations.

The change in thyroid test results of the 324 dogs between the first and the last examination is presented in Table 3.

**Table 3:** Outcome of 324 dogs in which follow-up data were available. The first and third columns provide the classification based on the first examination and the classification based on the last available examination, respectively.

First examination*	Number of dogs	Last examination*	Number of dogs (%)
Euthyroidism (group 2)	201	**group 1b**	**7 (3.5%)**
		group 2 (unchanged)	162** (80.6%)
		group 4	1 (0.5%)
		group 5	30 (14.9%)
		group 6	1 (0.5%)
Subclinical hypothyroidism (group 3)	6	**group 1a**	**4 (66.6%)**
		group 2	1 (16.7%)
		group 6	1 (16.7%)
TgAA-positive dogs with unremarkable thyroid function (group 4)	29	**group 1a**	**9 (31.0%)**
		group 2	10*** (34.4%)
		group 3	1 (3.5%)
		group 4 (unchanged)	7 (24.1%)
		group 5	1 (3.5%)
		group 6	1 (3.5%)
Consistent with non-thyroidal illness (group 5)	69	group 2	39 (56.5%)
		group 4	1 (1.5%)
		group 5 (unchanged)	28 (40.5%)
		group 6	1 (1.5%)
Unclassified (group 6)	19	**group 1a**	**4 (21.0%)**
		**group 1b**	**2 (10.5%)**
		group 2	10 (52.7%)
		group 4	1 (5.3%)
		group 6 (unchanged)	2 (10.5%)

* 1a = TgAA-positive hypothyroidism, 1b = TgAA-negative hypothyroidism, 2 = euthyroidism, 3 = subclinical hypothyroidism, 4 = TgAA-positive dogs with unremarkable thyroid function, 5 = potential nonthyroidal illness, 6 = unclassified

**TgAA were not reassessed in 78 dogs

***TgAA were not reassessed in 3 dogs

In total, 8% (26/324) of dogs developed hypothyroidism at a later point in time (group 1a or group 1b at the follow-up examination).

Overall, 62% (201/324) of the retested dogs had a normal thyroid profile at their initial examination (group 2). Of those, 80.6% (162/201) remained unremarkable. Almost 15 (14.9%) percent of dogs (30/201) with a normal thyroid profile showed thyroid test results consistent with nonthyroidal illness at their follow-up examination. Another 3.5 percent of dogs (7/201) with a normal thyroid profile developed TgAA-negative hypothyroidism. One dog with a normal thyroid profile demonstrated a TgAA-positive status at follow-up, and another dog depicted thyroid test results that were unclassified.

Subclinical hypothyroidism (group 3) was initially present in 1.9% (6/324) of the retested dogs. Four dogs with subclinical hypothyroidism had TgAA-positive hypothyroidism at the time of their final examination. One dog with subclinical hypothyroidism developed normal thyroid test results, while another dog had thyroid test results that could not be classified at follow-up. In this dog, the TSH level remained elevated, but the TgAA status had turned negative.

A positive TgAA status with unremarkable thyroid function (group 4) was detected in 9% (29/324) of the retested dogs at the first examination. Of those, 34.5% (10/29) exhibited a normal thyroid profile at follow-up. However, TgAA were not reassessed in 3 dogs. Thirty-one percent (9/29) developed TgAA-positive hypothyroidism. Seven dogs (7/29; 24.1%) remained TgAA-positive. One dog developed subclinical hypothyroidism, while another dog had a thyroid profile suggestive of nonthyroidal illness at its last examination. In a third dog, the test results could not be classified when retested. In this dog, the TgAA titer remained positive, and the TT4 concentration was below RI.

Test results suggestive of nonthyroidal illness (group 5) were initially present in 21.3% (69/324) of the retested dogs. Of those, 56.5% (39/69) tested unremarkably at follow-up. Twenty-eight dogs (28/69; 40.6%) remained in this group. One dog developed TgAA-positivity and another dog had thyroid test results that could not be classified at the follow-up examination. In the latter, the TT4 concentration remained low, but the TgAA status had become positive. In this group, no dog progressed to hypothyroidism.

In 5.9% (19/324) of the retested dogs, the thyroid test results could not be classified initially (group 6). More than half (52.6%; 10/19) of the dogs showed a normal thyroid profile at follow-up. In 8 of those 10 dogs, the TT4 concentration had been elevated at the first examination (6 dogs had been 1 year of age at that time), and in the other 2 dogs, the TSH concentration had been above RI. Four dogs (4/19; 21.1%) developed TgAA-positive hypothyroidism. In all 4 dogs, the TgAA titer had been positive in the first examination, while the TT4 concentration had been below RI in 3 dogs and above RI in 1 dog. By the time of their follow-up examination, 2 dogs (2/19; 10.5%) had progressed to TgAA-negative hypothyroidism. In both of those dogs, the TSH concentration had been elevated, but their TgAA status had been negative and the TT4 concentration had been within RI at the first examination. In another dog, where initially the TT4 level had been greater than the RI and a positive TgAA status had been present, the TT4 normalized to the RI at follow-up, but the positive TgAA status remained. Two dogs remained in group 6 (in both dogs, TSH had been elevated and remained elevated).

### Predictive value of TgAA

One hundred eighteen dogs were TgAA-positive at their first screening, of which 22.0% (26/118) were already hypothyroid. Follow-up examinations were available from 40 TgAA-positive dogs. Of those, 42.5% (17/40) developed hypothyroidism (Table 4): 66.7% with subclinical hypothyroidism (4/6), 31% with TgAA-positivity and an unremarkable thyroid function (9/29), and 80% with an initially positive TgAA status in group 6 (not classified, 4/5). Sensitivity, specificity, positive predictive value (PPV) and negative predictive value (NPV) of TgAA to predict the presence of hypothyroidism were 44.8%, 93.6%, 22.0% and 97.7%, respectively. Sensitivity, specificity, PPV and NPV of TgAA to predict development of hypothyroidism at a later time were 65.4%, 92.3%, 42.5% and 96.8%, respectively.

**Table 4:** Cross table for sensitivity, specificity, positive predictive value and negative predictive value of TgAA status at the initial screening and follow-up examination.

	Hypothyroid (n)	Not hypothyroid (n)	Total (n)
**Initial screening**			
TgAA positive	26	92	118
TgAA negative	32	1,351	1,383
**Follow-up**			
TgAA positive	17	23	40
TgAA negative	9	275	284

Only 3.2% (9/284) of dogs with negative TgAA status at the initial examination (201 dogs euthyroidism, 69 dogs nonthyroidal illness, 14 dogs unclassified) developed hypothyroidism later on. Those 9 dogs comprised 7 euthyroid dogs (group 2) and 2 unclassified dogs (group 6).

## Discussion

The aim of this study was to assess a possible predisposition towards hypothyroidism and TgAA-positivity in the Eurasian dog breed in Germany. Therefore we reviewed a breed surveillance program for hypothyroidism with measurement of TT4, TSH and TgAA of a German Eurasian breed club. The results indicate an overall remarkable prevalence of 3.9% for hypothyroidism and 7.9% for TgAA-positivity in this population.

Overall, it is challenging to cite a representative rate of hypothyroidism in the general dog population because certain breeds demonstrate a higher prevalence of hypothyroidism. In addition, there are difficulties in diagnosing the disease reliably in all cases based on laboratory measurements, as up to 40% of hypothyroid dogs have normal TSH concentrations [24, 42, 43]. A general prevalence of hypothyroidism of 0.2–0.8% was previously described in populations of dogs from referral centers [31, 47]. A 1.88% prevalence of hypothyroidism was reported at the University of California-Davis in 2013 based on review of electronic records [7]. In 2014, the prevalence of endocrinopathies in dogs attending primary-care veterinary practices in England was 1.9% [48]. A recent study analyzed a population of dogs in the vet hospital with signs of endocrinopathies in Punjab, India, between June 2018 and February 2020, including 20,108 dogs aged 1 year or older. The prevalence of hypothyroidism in this preselected population was approximately 0.2% (35 dogs) [8]. Considering that Eurasians in the current study underwent a breeding surveillance program and were overall young and healthy, the 3.9% prevalence rate can be considered high. In addition, the true prevalence in this study was probably underestimated. Dogs affected by nonthyroidal illnesses may have TT4 concentrations less than the RI, but their TSH concentrations are more likely to remain within the RI [49]. However, as 25–40% of dogs with hypothyroidism have a TSH concentration within or less than the RI [42, 43], hypothyroid dogs may also display the same laboratory combination and can be indistinguishable from dogs with nonthyroidal illnesses. Therefore, it is possible that dogs assessed as potential nonthyroidal illness (group 5) included dogs which were in the final stage of autoimmune thyroiditis as described by Graham *et al*. [3, 13]. Adding 25% to dogs with hypothyroidism (group 1a and group 1b), this would sum up to a prevalence of hypothyroidism of 5.1%. In addition, TT4 concentrations of dogs with subclinical hypothyroidism (group 3) and unclassified results (group 6) could have been elevated by the presence of T4 autoantibodies [3]. Apart from hypothyroidism (3.9%), 21.9% of the Eurasians had other abnormalities in thyroid parameters (groups 3, 4, 5 and 6). Overall, dogs with subclinical hypothyroidism (group 3), isolated TgAA-positivitiy (group 4) and unclassified thyroid test results (group 6) already had evidence of thyroid dysfunction indicated by either increased TSH concentrations or increased TT4 concentrations and/or presence of TgAA and could also have developed hypothyroidism in the long term. This was demonstrated by the 17 dogs of groups 3, 4 and 6 that were diagnosed as hypothyroid during follow-up examinations.

The overall prevalence of hypothyroidism in Eurasians is comparable to the prevalence noted in other predisposed breeds, however, breeds with an even higher prevalence have been reported, but diagnostic criteria varied between studies. In Beagles, 15.9% were reported to be hypothyroid at their time of death in a closed breeding colony [50]; however, inbreeding was a concern in this population. The diagnosis of hypothyroidism was based on clinical observations, necropsy examination, histopathologic interpretations and additional laboratory data [50]. In a cohort study by Ferm *et al*. (2009), 7.2% of Giant Schnauzers and 3.2% of Hovawarts were considered hypothyroid. An additional 9.3% of Giant Schnauzers and 9.5% of Hovawarts had laboratory findings suggestive of CLT or hypothyroidism (elevated TSH and/or positive TgAA titer) but had not developed clinical signs. The diagnosis of hypothyroidism or CLT was based on elevated TSH concentrations and/or positive TgAA status accompanied by information gained from a dog owner questionnaire [1]. Therefore, in contrast to the present study, TT4 measurements were not included in the diagnosis in the study by Ferm *et al*; in addition, an elevated TSH concentration was considered diagnostic for hypothyroidism, although elevated TSH concentrations could also occur in euthyroid dogs [42, 51] or dogs with nonthyroidal illness [44, 49, 52].

Published literature to date suggests that hypothyroidism is a disease of middle aged dogs [2, 31, 47, 53]. Specifically, hypothyroidism had been diagnosed in the Gordon Setter at median of 6.4 years, in the English Setter at 7.7 years [2], in Giant Schnauzers and Hovawarts at 6–7 years [1], in Beagles at 2.7–6.8 years [54] and in a mixed population of hypothyroid dogs at a mean age of 7.6 years [51]. Dixon *et al*. noted that dogs can be affected at any age, but the disease is rarely identified before two years of age [47]. High-risk breeds have been reported to have an earlier onset of hypothyroidism [55, 56]. The present study shows that hypothyroidism is already diagnosed frequently in two- to four-year-old Eurasians. This finding confirms that screenings should be performed at regular intervals in young dogs of the Eurasian dog breed. However, TgAA-negative hypothyroidism in Eurasians was diagnosed in older dogs as well (up to the age of 11 years). The literature suggests that previously high TSH serum concentrations decrease in hypothyroid dogs over time [57], so hypothyroidism was possibly not detected in some of the older dogs in this study.

Females were more frequently affected by TgAA-negative hypothyroidism (group 1b) and subclinical hypothyroidism (group 3) (p<0.05 in both cases). As Eurasians in this study were generally young and given the lack of a statistically significant gender difference in groups 1a (TgAA-positive hypothyroidism) and 4 (TgAA-positivity with unremarkable thyroid function), female dogs might progress through the stages described by Graham *et al*. [3, 13] faster than males. However, the data of the present study cannot clarify this notion. There have been contradictory reports on whether sex and neutering status influence the likelihood of developing hypothyroidism [31, 47, 50, 55, 56, 58–60]. To date, a clear consensus on a sex-based predisposition is not available. There were also statistically significant differences in regard to gender in euthyroid dogs (group 2) and dogs with potential nonthyroidal illness (group 5). The overrepresentation of males in group 5 might indicate that their TT4 concentrations have a greater tendency to decrease in the case of nonthyroidal illness.

In this study, 7.9% (118/1,501) of all dogs included and also 45% (26/58) of hypothyroid dogs had a positive TgAA status. The presence of TgAA could indicate CLT as described by Graham *et al*. [3], however, the term is technically based on histopathology, although TgAA status may be used as indirect evidence of the presence of CLT [61]. The authors of this study were reluctant to use the term CLT as histology was not performed and the TgAA status was assessed only once in the majority of dogs. However, the given prevalence of hypothyroidism and the high predictive value of TgAA, which was indicated by our data, suggest that TgAA-positivity truly resembled presence of CLT in this population of Eurasians. As a consequence, CLT seems to be an important cause of hypothyroidism in the Eurasian dog breed. In general, approximately 50% of hypothyroid dogs test positive for TgAA [13, 17, 20, 62–64]. Graham *et al*. reported that specific breeds are predominantly affected by TgAA-positive hypothyroidism [3, 13]. For example, 84% of hypothyroid English Setters had TgAA, whereas only 16% of hypothyroid Dachshunds were TgAA positive. This finding may indicate a different pathogenesis of thyroid dysfunction and different progression rates of the diseases [3, 13]. Graham *et al*. stated that the high prevalence of TgAA among different dog breeds supports the genetic basis of CLT [13] and serves as a marker of thyroiditis [3].

In the present study, the Eurasians with TgAA-positive hypothyroidism were approximately 2 years younger than those with TgAA-negative hypothyroidism. It is very well possible that some of the TgAA-negative hypothyroid dogs had previously been TgAA-positive and antibodies had disappeared over time, which would explain the older age of these dogs. This observation is also consistent with the literature, as studies have shown that TgAA-positive hypothyroid dogs are younger (mean age 6.6 years) than TgAA-negative hypothyroid dogs (mean age 8.1 years) [3, 55].

In the present study, a previously proposed classification scheme was used to describe progression of TgAA-positivity to functional thyroid failure. This scheme was based on the four stages described by Graham *et al*. [3, 13]. Although our classification of the different groups was artificial to some extent, in retrospect this scheme seems adequate, as the follow-up data of dogs truly showed progression from thyroiditis or subclinical hypothyroidism to hypothyroidism. In dogs with subclinical hypothyroidism, 4/6 dogs progressed to TgAA-positive hypothyroidism. In dogs with TgAA-positivity, 9/29 dogs progressed to TgAA-positive hypothyroidism and another dog progressed from TgAA-positivity to subclinical hypothyroidism. In dogs with unclassified thyroid test results, 6/19 dogs progressed to hypothyroidism (4 TgAA-positive and 2 TgAA-negative hypothyroidism).

Follow-up examination of 324 dogs revealed two important aspects. First, 3.5% (7/201) of dogs that had an unremarkable first test result developed hypothyroidism at a later examination. This finding indicates that hypothyroidism can still develop later on even if no abnormalities were previously detected. Second, the predictive value of TgAA to be hypothyroid or to develop hypothyroidism was quite high in the present study. Specifically, 22% (26/118) of dogs with a positive TgAA status were already diagnosed hypothyroid, and 42.5% (17/40) of dogs with a positive TgAA status, which were not initially classified as hypothyroid, subsequently developed hypothyroidism. The latter percentage may have been even higher with a longer follow-up. Graham *et al*. reported that 1 of 5 TgAA-positive dogs developed progressive thyroid dysfunction within one year, and 1 of 20 TgAA-positive dogs were already hypothyroid [3]. Our findings support the findings of Graham et al. and indicate an even higher predictive value of TgAA in the Eurasian dog breed. On the other hand, only 3.2% (9/284) of dogs with absence of TgAA at the initial examination subsequently developed hypothyroidism, indicating a high NPV when no TgAA are present. In a previous investigation from Germany, 156 TgAA-positive dogs with nonthyroidal illnesses were followed-up by the University of Munich for two years [65]. No clinical signs of thyroid dysfunction were noted at initial examination. Of these TgAA-positive dogs, 22.5% developed overt hypothyroidism within two years, 13.5% had a TT4 concentration less than the RI, and 6.4% had a TSH concentration greater than the RI at follow-up examination but no clinical signs of hypothyroidism were observed. In 14.1% of TgAA-positive dogs, the TgAA titer decreased [65]. As mentioned above, in the present study, an even higher percentage (42.5% of the repeatedly tested TgAA-positive dogs) developed hypothyroidism on follow-up, which highlights that TgAA are an important screening parameter. Consequently, the presence of TgAA should be taken seriously and regarded as a clinically relevant marker of thyroid disease. Therefore, thyroid function testing, including the measurement of TgAA titers, should be performed at various follow-up time points, especially in predisposed breeds.

In this study, only TgAA were measured, and other autoantibodies were not tested. However, TgAA seem to be the most important anti-thyroidal autoantibodies in dogs (in 51% of hypothyroid dogs) followed by antibodies against T3 (T3AA) (28%) and T4 (T4AA) (8%) [3, 13, 17, 20, 66]; both T3AA and T4AA were found in 6% of hypothyroid dogs [3]. Of hypothyroid dogs, 26% have only TgAA but no thyroid hormone antibodies (i.e. T3AA and T4AA). In contrast, only 5% of hypothyroid dogs had antibodies against thyroid hormones but no TgAA [3].

Vaccination of dogs can induce various autoantibodies [67]. Thyroglobulin autoantibodies (TgAA) can be borderline positive in the months after vaccination [68]. However, this was not demonstrated in all studies [67] and no association between vaccinations and thyroiditis at postmortem examination was found [69]. To minimize any effects of vaccination, breeders of this study were advised to schedule blood sampling as late as possible after vaccination. However, this was only a recommendation and it was not evident from the data provided, how much time had elapsed between the last vaccination and performance of the thyroid panel.

Several dog breeds have different reference ranges for TT4 and TSH. This is well documented in sighthounds (e.g., Greyhounds, Sloughis and Salukis). These dogs have lower TT4 and fT4 reference ranges than nonsighthound dogs [70–72]. In the Samoyed breed, which also contributed to the gene pool of Eurasians, TT4 serum concentrations matched the reported reference range for dogs [73]. Future studies should focus to establish breed-specific-reference ranges in Eurasian dogs.

One limitation of this study is the retrospective analysis of the data. Another limitation of this study was that the diagnosis of hypothyroidism was only established by means of laboratory test results and that the dogs were not clinically examined. Hypothyroidism in this study was based on a combination of a low TT4 concentration and a high TSH concentration. A sensitivity of 86.7% and a specificity of 92.2% for diagnosing hypothyroidism were reported for the TT4/TSH ratio [51]. In hypothyroid dogs, TSH can be within the reference range [42–44, 51, 52], as noted previously. Therefore, potentially not all hypothyroid dogs in this study were identified, leading to an underestimation of hypothyroidism. The lack of a breed-specific reference range for TT4 and TSH can be referred to as further limitation.

Unfortunately, only 21.6% (324/1,501) of dogs were retested, and a negative TgAA status was not always reassessed. The breeding club provided recommendations to the owners that participating dogs undergo retesting, however, owners decided whether their dog participated in regular and complete screenings (retesting of TT4, TSH and TgAA status). In dogs, in which TgAA were measured, a titer was not reported for all dogs. Therefore, analysis was only possible based on the categorization of “positive” versus “negative”. The impact of the TgAA titer level itself on the development of hypothyroidism was not analyzed. As mainly young Eurasians were included in this study, longer follow-up times and retesting at regular intervals in more individuals would have been desirable and could have generated more distinct results.

## Conclusion

This study indicated that the Eurasian dog breed is predisposed to hypothyroidism. The relevant proportion of TgAA-positive dogs suggests an immune-mediated mechanism of the disease. Thyroglobulin autoantibodies (TgAA) are a valuable marker to predict the occurrence of hypothyroidism. This marker should be implemented in breeding programs and positive titers should be followed carefully. Therefore, breeding clubs should encourage owners to keep their dogs involved in screening programs including the measurement of TgAA, and such programs should be established in any dog breed where hypothyroidism and CLT are suspected to be heritable diseases. Further investigations with longitudinal studies are warranted.

The study also showed that careful surveillance programs might help to discover inherent predispositions for a disease in a population of breeding dogs. This practice should be acknowledged as a very responsible and proactive management technique by the Eurasian breeding club.

## Supporting information

S1 Fig Age distribution in groups 1 (all dogs with hypothyroidism), 2 (euthyroid), 3 (subclinical hypothyroidism), 4 (TgAA-positive dogs with unremarkable thyroid function), 5 (potential nonthyroidal illness) and 6 (unclassified). (TIF)

S2 Fig Prevalence of males and females in the different groups (group 1 = all dogs with hypothyroidism, group 2 = euthyroid, group 3 = subclinical hypothyroidism, group 4 = TgAA-positive dogs with unremarkable thyroid function, group 5 = potential nonthyroidal illness, and group 6 = unclassified). (TIF)

S3 Fig Distribution of TT4 concentrations in groups 1 (all dogs with hypothyroidism), 2 (euthyroid), 3 (subclinical hypothyroidism), 4 (TgAA-positive dogs with unremarkable thyroid function), 5 (potential nonthyroidal illness) and 6 (unclassified). (TIF)

S4 Fig Distribution of TSH concentrations in groups 1 (all dogs with hypothyroidism), 2 (euthyroid), 3 (subclinical hypothyroidism), 4 (TgAA-positive dogs with unremarkable thyroid function), 5 (potential nonthyroidal illness) and 6 (unclassified). (TIF)

S1 File Raw data table of the 1,501 dogs analyzed. (XLSX)

S2 File Raw data table of 324 dogs that were re-tested. (XLSX)
