# Supplementary figures and images for: Laboratory indicators of hypothyroidism and TgAA-positivity in the Eurasian dog breed
Source: PLoS One. 2023 Jan 24;18(1):e0280906. doi: 10.1371/journal.pone.0280906 (PMC9873187; doi:10.1371/journal.pone.0280906)

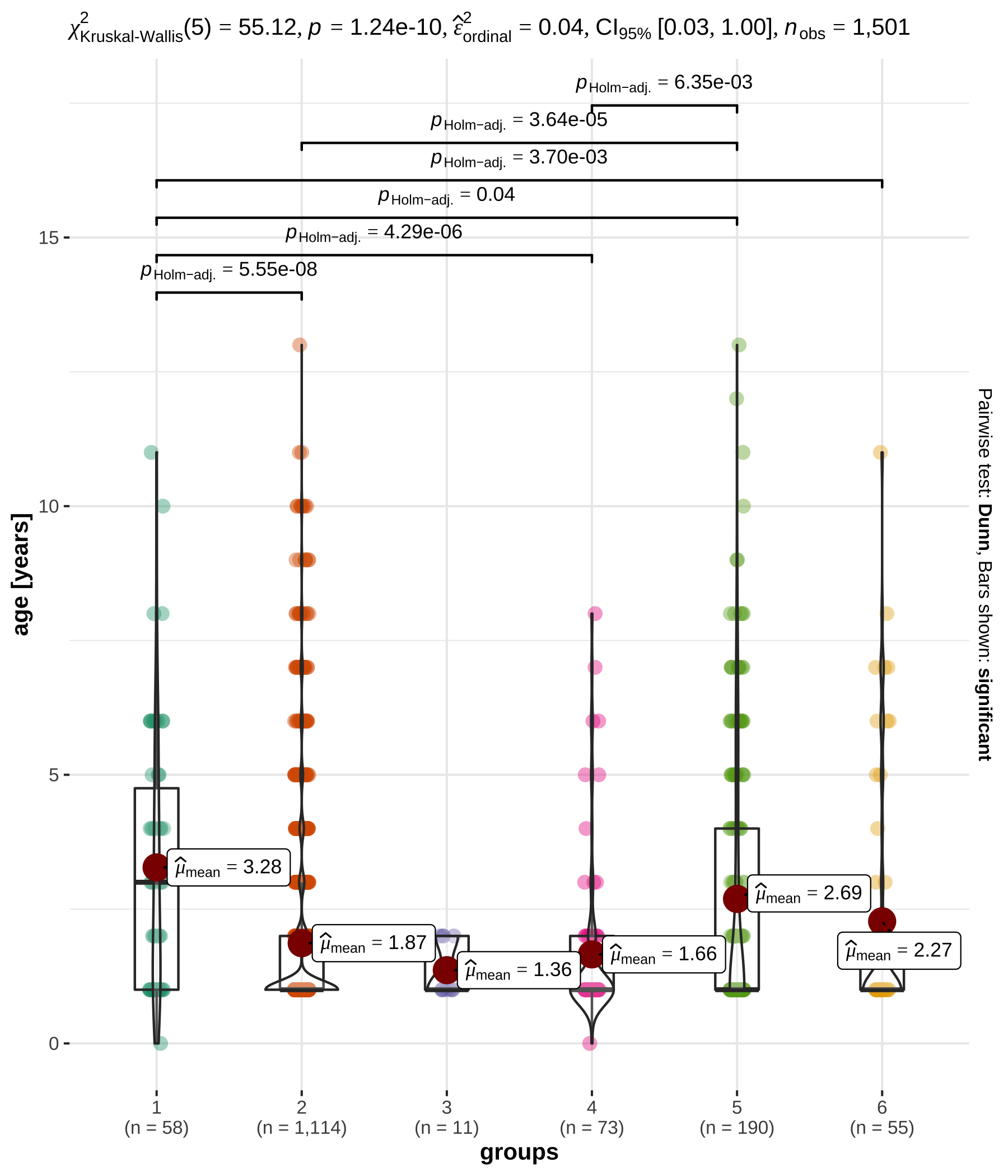

Supplement: S1 Fig — (TIF) [file pone.0280906.s001.tif]

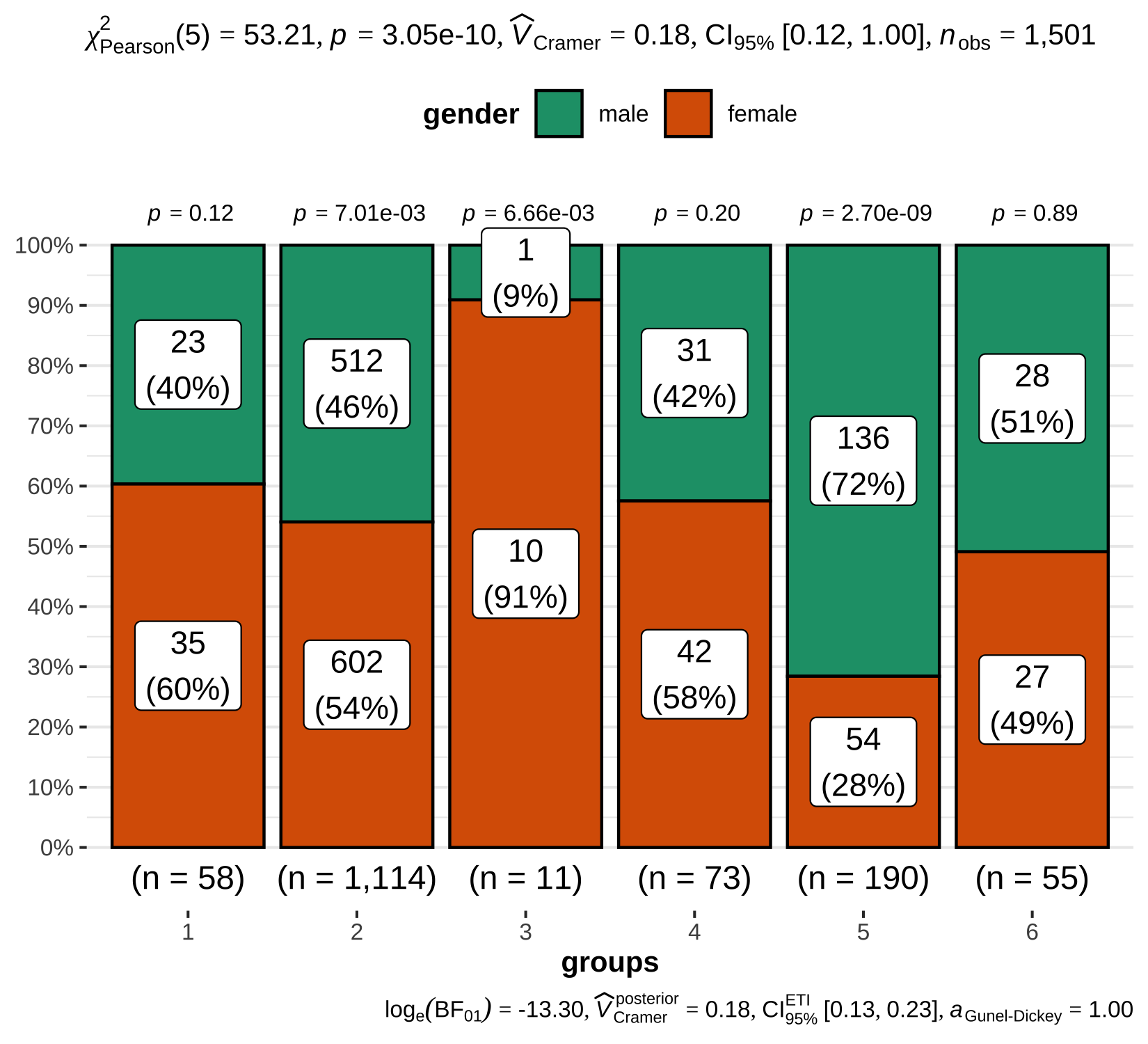

Supplement: S2 Fig — (TIF) [file pone.0280906.s002.tif]

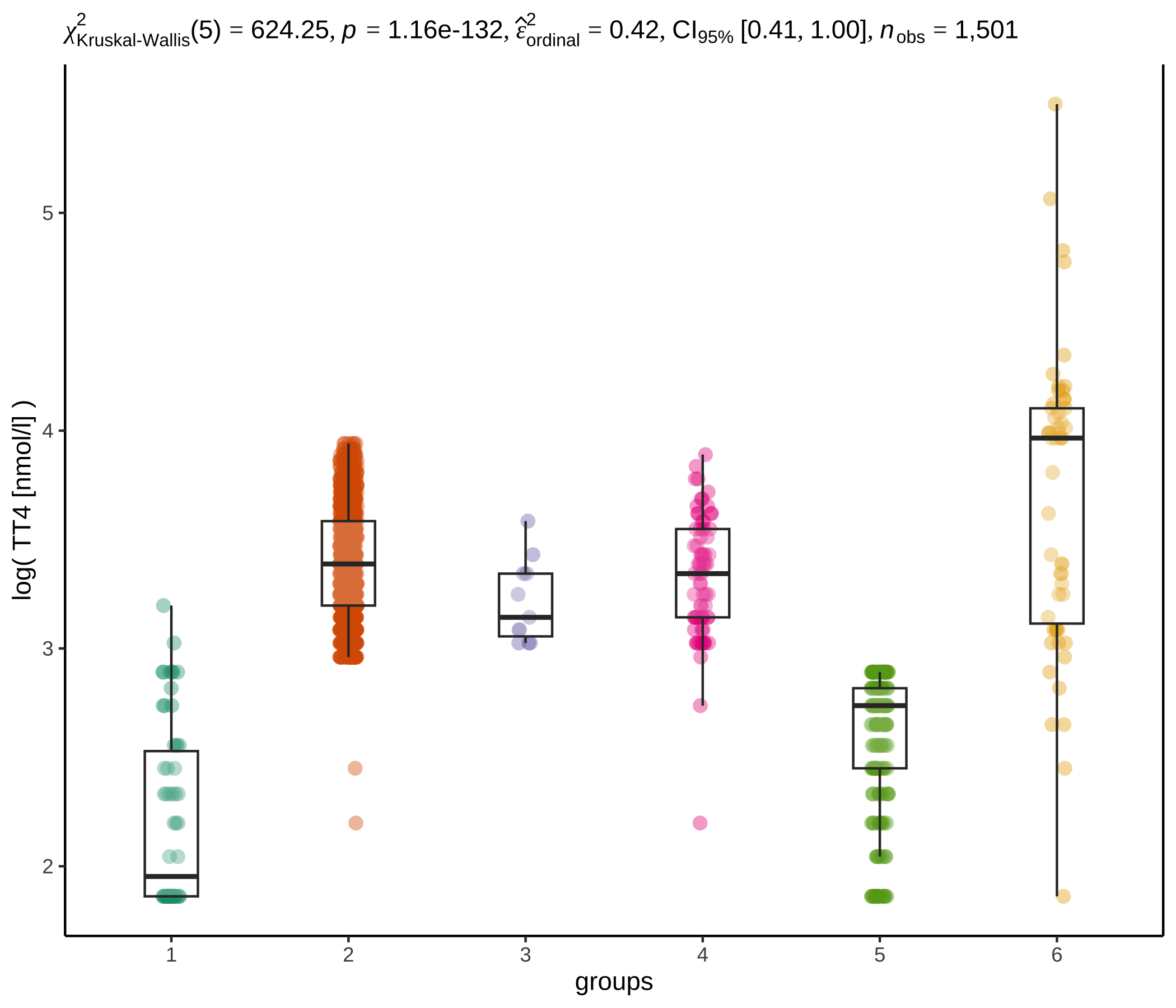

Supplement: S3 Fig — (TIF) [file pone.0280906.s003.tif]

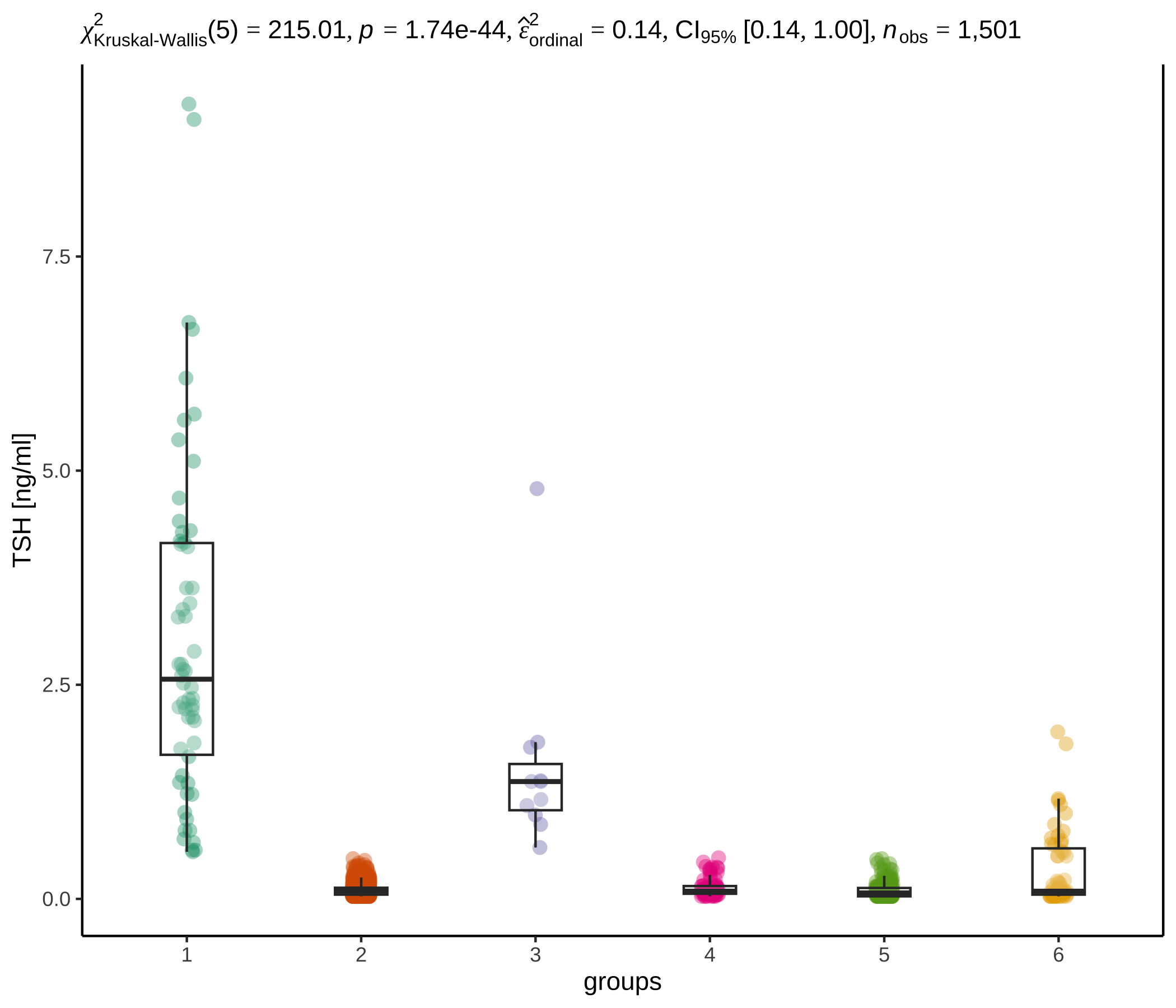

Supplement: S4 Fig — (TIF) [file pone.0280906.s004.tif]
